# Effectiveness of iguratimod as monotherapy or combined therapy in patients with rheumatoid arthritis: a systematic review and meta-analysis of RCTs

**DOI:** 10.1186/s13018-021-02603-2

**Published:** 2021-07-16

**Authors:** Chao-Jun Hu, Li Zhang, Shuang Zhou, Nan Jiang, Jiu-Liang Zhao, Qian Wang, Xin-Ping Tian, Xiao-Feng Zeng

**Affiliations:** Department of Rheumatology, Peking Union Medical College Hospital; National Clinical Research Center for Dermatologic and Immunologic Diseases (NCRC-DID); Key Laboratory of Rheumatology and Clinical Immunology, Ministry of Education, Peking Union Medical College & Chinese Academy of Medical Sciences, No. 1 Shuaifuyuan, Dongcheng District, Beijing, 100730 China

**Keywords:** Iguratimod, Monotherapy, Combined therapy, Rheumatoid arthritis, Meta-analysis

## Abstract

**Background:**

This study aims to evaluate the efficacy and safety of the iguratimod (IGU) as monotherapy or combined therapy in patients with rheumatoid arthritis (RA) by using meta-analysis.

**Methods:**

We searched Medline, EMBASE, Cochrane library, CNKI, Wanfang medical network from initial to 30 June, 2020, for randomized clinical trials (RCTs). Two authors independently screened the studies via reading the title, abstract, and full text. The risk of bias in individual studies was assessed using the Cochrane Risk of Bias tool. STATA 12.0 was used for pooled analysis of all included studies.

**Results:**

A total of 23 RCTs were included in this analysis. Meta-analysis showed that patients in the IGU monotherapy or combined therapy group had significantly higher ACR20 (OR = 1.97, 95% CI 1.29 to 3.00, *P* = 0.002), lower DAS28-CRP (SMD = −3.49, 95% CI −5.40 to −1.58, *P* < 0.001) and DAS28-ESR (SMD = −2.61, 95% CI −3.64 to −1.57, *P* < 0.001), as well as shorter duration of morning stiffness (SMD = −2.06, 95% CI −2.86 to −1.25, *P* < 0.001) and lower HAQ score (SMD = −0.91, 95% CI −1.61 to −0.21, *P* = 0.011), than those received other disease-modifying antirheumatic drugs (DMARDs) monotherapy (primarily comprising methotrexate). For the safety profile, IGU monotherapy had similar risks for gastrointestinal reactions (*P* = 0.070), leucopenia (*P* = 0.309), increment in transaminase (*P* = 0.321), increase of ALT (*P* = 0.051), and liver damage (*P* = 0.182) to methotrexate monotherapy, and IGU combined with other DMARDs therapy did not increase the risks of these AEs (*P* > 0.05).

**Conclusions:**

Our evidence suggests that IGU is effective and tolerant as monotherapy or combined therapy especially with methotrexate in patients with active RA. IGU may be regarded as a potential alternative to methotrexate, and a preferable choice when combined with other DMARDs for the treatment of RA.

**Supplementary Information:**

The online version contains supplementary material available at 10.1186/s13018-021-02603-2.

## Background

Rheumatoid arthritis (RA) is a chronic, inflammatory autoimmune disease, which is characterized by inflammation of synovial joints and cartilage erosion, leading to functional disability and increased risk of premature death [1]. Despite there being abundant drugs for treating RA, including conventional synthetic disease-modifying antirheumatic drug (csDMARD), biologic DMARD (bDMARD), and targeted synthetic DMARD (tsDMARD), a considerable number of patients (about 20-30%) still do not achieve remission or at least low disease activity [2]. The use of bDMARD and tsDMARD often result in rapid and sustained clinical remission for the majority of patients, while are also associated with the risk of adverse events (AEs), such as prolonged immunosuppression, infection, and economic implications [3, 4]. Hence, csDMARD, ideally methotrexate, remains the basis of RA treatment in clinic, especially in low-income countries. According to the 2019 EULAR recommendations, csDMARD are primarily used as induction and maintenance therapy for patients who receive initial treatment or have inadequate response to initial csDMARD monotherapy to reach the target of sustained remission [5]. For patients with persistent remission after bDMARD or tsDMARD therapy, tapering bDMARD or tsDMARD should be considered, and may be maintained with csDMARD [6]. Therefore, csDMARD plays a pivotal role in RA treatment.

Iguratimod (IGU), a novel small-molecule DMARD which has a unique mechanism of action compared with that of other DMARDs. As a novel immunomodulator, IGU act simultaneously on T and B lymphocytes to regulate the balance of immune cells and cytokines, such as T-bet, IL-17, STAT3, Bcl6, IL-21, IFN-γ, TNF-α, and IL-17A [7]. IGU also has an anti-inflammatory role via inhibiting canonical inflammation-associated signaling pathways, such as NF-κB [8–11] and IL-17R pathway [12, 13]. Moreover, IGU demonstrated anabolic effects on bone metabolism. IGU can increase the expression of osterix and Dlx5 to promote osteoblast differentiation [14], and inhibiting the expression of MMP-1, MMP-3 [15], and RANKL/OPG [16, 17] to suppress bone resorption.

There are amounts of studies on IGU monotherapy or combined therapy. Previous studies demonstrated that IGU monotherapy was non-inferior to methotrexate (MTX) in efficacy, but more tolerant than MTX [18]. In addition, several randomized controlled trials (RCTs) indicated that IGU plus MTX has also achieved better effectiveness than MTX monotherapy in patients with RA [19, 20]. Consequently, in the 2015 Asia Pacific League of Associations of Rheumatology (APLAR) guideline, iguratimod was suggested as first-line treatment in the condition that patients who cannot tolerate MTX in some Asia-Pacific countries [21]. Moreover, the latest version of 2018 APLAR guideline also recommends the application of iguratimod as the 2015 version [22]. Iguratimod may be added to methotrexate to enhance efficacy when response to MTX monotherapy is inadequate [23].

A recently published meta-analysis evaluated the efficacy and safety of iguratimod monotherapy over other DMARDs [24]. Although amounts of evidences of IGU combined therapy have been accumulated, the efficacy and safety of IGU combined therapy have been poorly estimated. Therefore, this systematic review aims to comprehensively assess the efficacy and safety of IGU therapy (IGU monotherapy and combined therapy) for RA to provide more guidance of IGU use in the future.

## Methods

### Literature search

Two researchers searched the Medline, EMBASE, Cochrane library, CNKI, Wanfang medical network from initial to 30 June, 2020, and they reviewed the title, abstract, and even the full text at the same time to determine whether the study met the inclusion criteria independently. Search strategy for the PubMed database is given as follows: ((Iguratimod OR T-614 OR IGU) AND (rheumatoid arthritis OR RA)) AND (English[Language]).

### Inclusion and exclusion criteria

The following criteria were adopted: (1) randomized control trials (RCTs) that evaluate the efficacy and safety of IGU monotherapy and IGU combined MTX therapy with MTX as a comparator drug, and the efficacy of IGU plus other DMARDs compared with other DMARDs monotherapy in treating patients with RA; (2) patients receiving a diagnosis of RA according to the 1987 American College of Rheumatology (ACR) or 2010 ACR/European League against Rheumatism (EULAR) RA classification criteria [25, 26]; and (3) there are no mixed intervention in the test group and control group. (4) One or more of the following outcomes were reported to allow data on to be extracted: American College of Rheumatology (ACR) 20, Disease Activity Score in 28 joints—C-reactive protein (DAS28-CRP), DAS28-erythrocyte sedimentation rate (ESR), Health Assessment Questionnaire (HAQ) score, and duration of morning stiffness.

We excluded the following articles: (1) incomplete or duplicative data; (2) Chinese medicine in the combination group and/or the control group; (3) the control group was intervention with placebo; (4) patients with both RA and cancer, renal dysfunction, or other complications; (5) case reports, reviews, etc.

### Data extraction and quality assessment

Two researchers read the full text of selected eligible studies at the same time, and extracted the following data from each study, including the author, title, year, design, outcome, and other specific values in the study. Methodological quality of the included studies was assessed using Cochrane Risk of Bias tool (version 5.1.0, updated in March 2011) which is developed for assessing the quality of RCTs. The Cochrane Risk of Bias tool included 6 domains: selection, performance, detection, attrition, reporting, and other bias [27].

### Statistical analysis

The statistical analysis was performed by authors using STATA 12.0 (StataCorp LP, College Station, Texas). Heterogeneity among the included studies was tested, and the size of heterogeneity was determined according to Cochran’s Q statistic and the *I*^2^ statistic. Low heterogeneity was defined as 25% < *I*^2^ < 50%; moderate heterogeneity was defined as *I*^2^ ≥ 50% and high heterogeneity was defined as *I*^2^ ≥ 75%. According to the heterogeneity, fixed or random effect models were used between different studies. When there was no heterogeneity in the included studies, the fixed effects model was used for meta-analysis; otherwise, the random effects model was used, and *P* < 0.05 was considered statistically significant. In addition, sensitivity analysis was performed to ensure the robustness of results, and the summarized odd ratio (OR) or standard mean difference (SMD) was analyzed with the omission of one study at a time to detect whether the overall results were strongly affected by a specific study. Publication bias was evaluated through Egger’s linear regression and visual inspection of funnel plots.

## Results

### Study selection

**Fig. 1:**
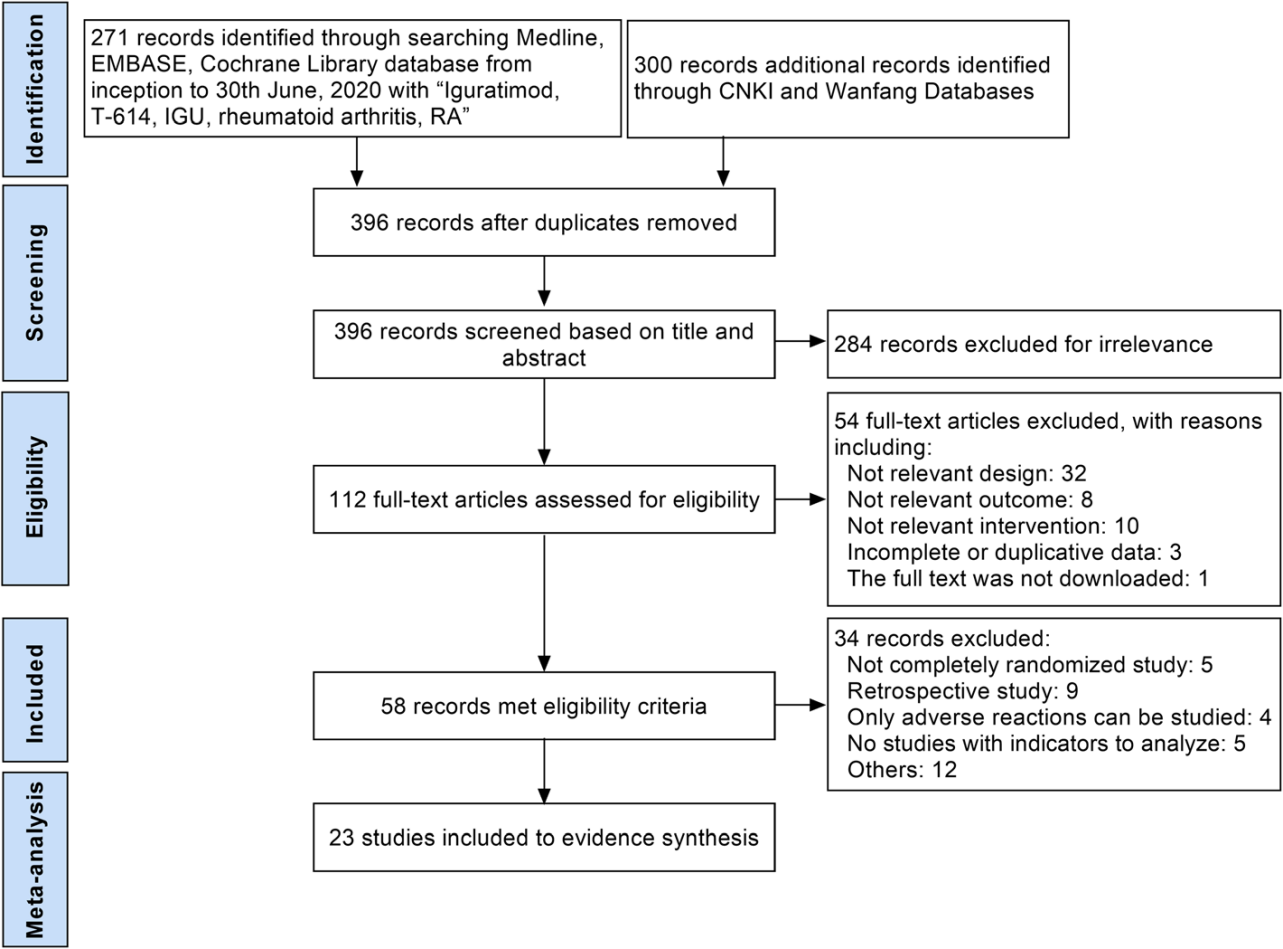
Study selection flow diagram

We identified 571 citations and the detailed article search process was presented in Fig. 1. According to the inclusion and exclusion criteria, 23 selected articles involving 2533 patients were included in this analysis finally [18–20, 28–47]. Three RCTs compared IGU monotherapy versus MTX monotherapy, 18 RCTs compared IGU plus MTX versus MTX monotherapy (7 RCTs of them compared IGU plus MTX, MTX monotherapy and IGU monotherapy), 1 RCT compared IGU plus leflunomide versus leflunomide monotherapy, and 1 RCT compared IGU plus etanercept versus etanercept monotherapy. The duration of treatment ranged from 12 to 68 weeks, most of them were 24 weeks. ACR20 response, DAS28-CRP, DAS28-ESR, HAQ score, duration of morning stiffness, and adverse events were used to measure outcomes in 8, 4, 8, 7, studies respectively. Characteristics of included studies were listed in Table 1.

**Table 1:** Characteristics of included studies

Study	Participants, ***n***	RA type	Intervention	Duration	Outcomes
Xia 2016	150	Active RA	IGU+MTX vs. IGU vs. MTX	24 weeks	ACR20, ACR50, ACR70, DAS28-ESR, DAS28-CRP, duration of morning stiffness, HAQ, adverse events.
Zhao 2017	96	Active RA	IGU+MTX vs. IGU vs. MTX	24 weeks	ACR20, ACR50, ACR70, DAS28-CRP, HAQ, adverse events.
Cao 2018	90	Active RA	IGU+MTX vs. IGU vs. MTX	24 weeks	DAS28-CRP, HAQ
Xu 2015	110	Active RA	IGU+MTX vs. IGU vs. MTX	52 weeks	Duration of morning stiffness, adverse events.
Zhao 2016	90	Active RA	IGU+MTX vs. IGU vs. MTX	24 weeks	ACR20, ACR50, ACR70, adverse events.
Xiong 2015	86	Active RA	IGU+MTX vs.IGU vs. MTX	24 weeks	DAS28-ESR, adverse events.
Lv 2014	131	Active RA	IGU+MTX vs. IGU vs. MTX	24 weeks	DAS28-ESR, DAS28-CRP, duration of morning stiffness, HAQ
Duan 2015	60	Active RA	IGU+MTX vs. MTX	24 weeks	ACR20, ACR50, ACR70, DAS28, HAQ, adverse events.
Ishiguro 2013	252	Active RA	IGU+MTX vs. MTX	24 weeks	ACR20, ACR50, ACR70, HAQ, adverse events.
Ren 2017	82	Active RA	IGU+MTX vs. MTX	26 weeks	Duration of morning stiffness, adverse events.
Wang 2017	120	Active RA	IGU+MTX vs. MTX	68 weeks	DAS28-ESR, adverse events.
Bai 2015	100	Active RA	IGU+MTX vs. MTX	24 weeks	ACR20, ACR50, adverse events.
Xie 2018	120	Refractory RA	IGU+MTX vs. MTX	17 weeks	DAS28-ESR, adverse events.
Wang 2016	87	Refractory RA	IGU+MTX vs. MTX	24 weeks	DAS28, adverse events.
Meng 2016	60	Refractory RA	IGU+MTX vs. MTX	16 weeks	DAS28-ESR, adverse events.
Chen 2018	120	Active RA	IGU+MTX vs. MTX	24 weeks	Duration of morning stiffness, adverse events.
Xu 2017	83	Active RA	IGU+MTX vs. MTX	52 weeks	DAS28, adverse events.
Xiong 2020	102	Active RA	IGU+MTX vs. MTX	24 weeks	Duration of morning stiffness, adverse events.
Lu 2009	326	Active RA	IGU vs. MTX	24 weeks	ACR20, ACR50, ACR70, duration of morning stiffness, HAQ, adverse events.
Yang 2017	60	Active RA	IGU vs. MTX	12 weeks	DAS28-ESR, duration of morning stiffness.
Hu 2014	40	Active RA	IGU vs. MTX	24 weeks	ACR20, DAS28-ESR, adverse events.
Dai 2019	108	Active RA	IGU+Leflunomide vs. Leflunomide	12 weeks	DAS28-3, duration of morning stiffness, adverse events.
Li 2018	60	Refractory RA	IGU+ Etanercept vs. Etanercept	12 weeks	DAS28-ESR, adverse events.

### Quality assessment

**Fig. 2:**
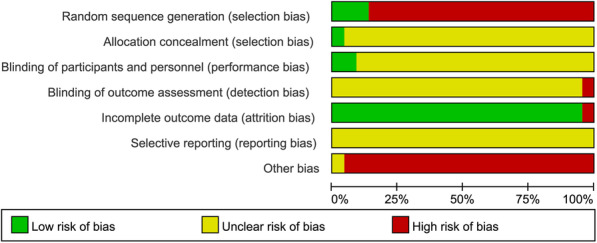
Methodological quality of included studies in the meta-analysis

As displayed in Fig. 2, random allocation was reported in all included studies, but only a few studies adopted random number table. Allocation concealment, blinding of participants and personnel, and blinding of outcome assessment for most studies were assessed as unclear risk due to the related data was not described. All studies had low risk of incomplete outcome, while the risk of other bias was assessed as high.

### Efficacy

**Fig. 3:**
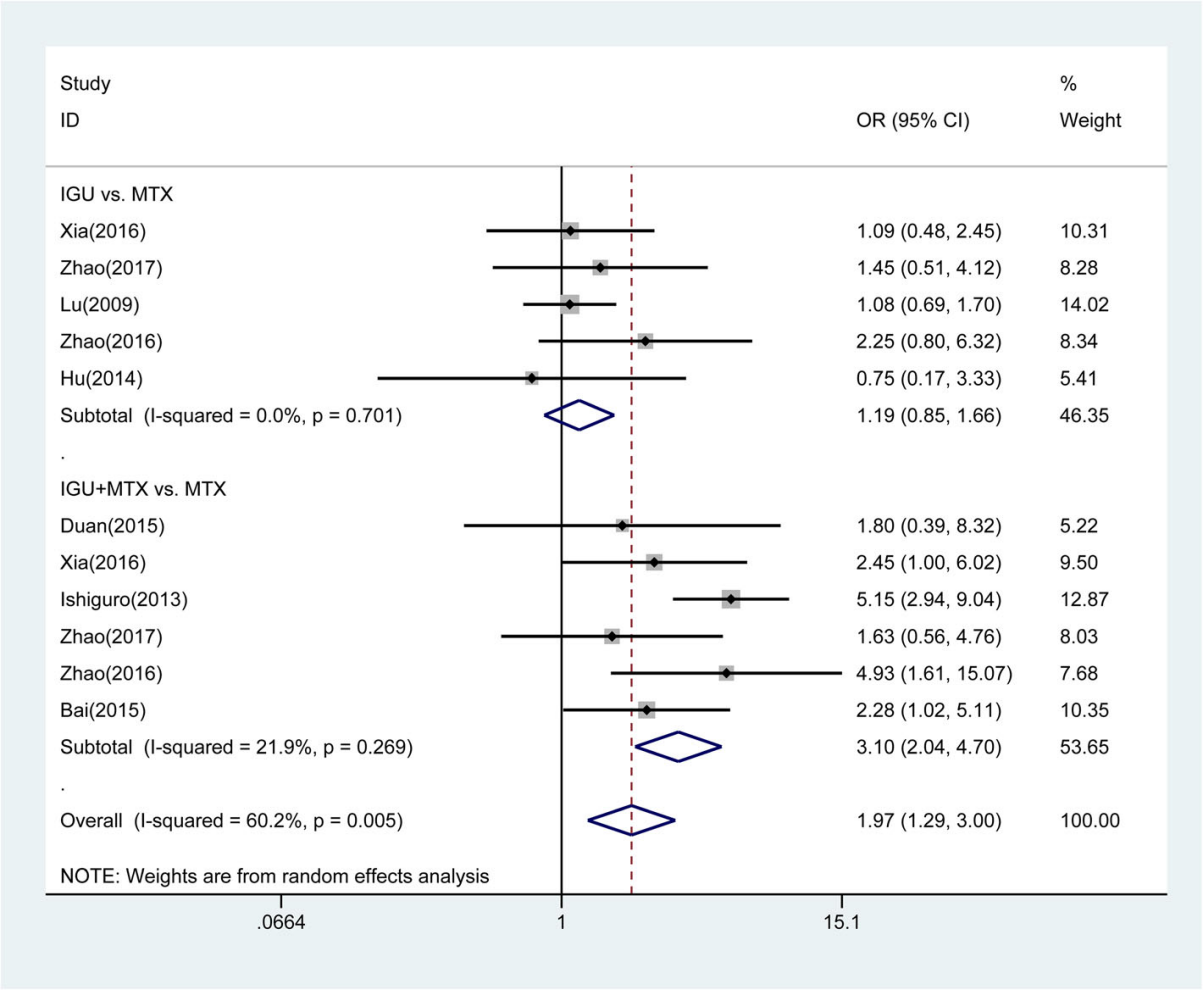
Comparison of ACR20 between IGU therapy and other DMARDs monotherapy (primarily comprising MTX). IGU, iguratimod; MTX, methotrexate; DMARDs, disease-modifying antirheumatic drugs

**Fig. 4:**
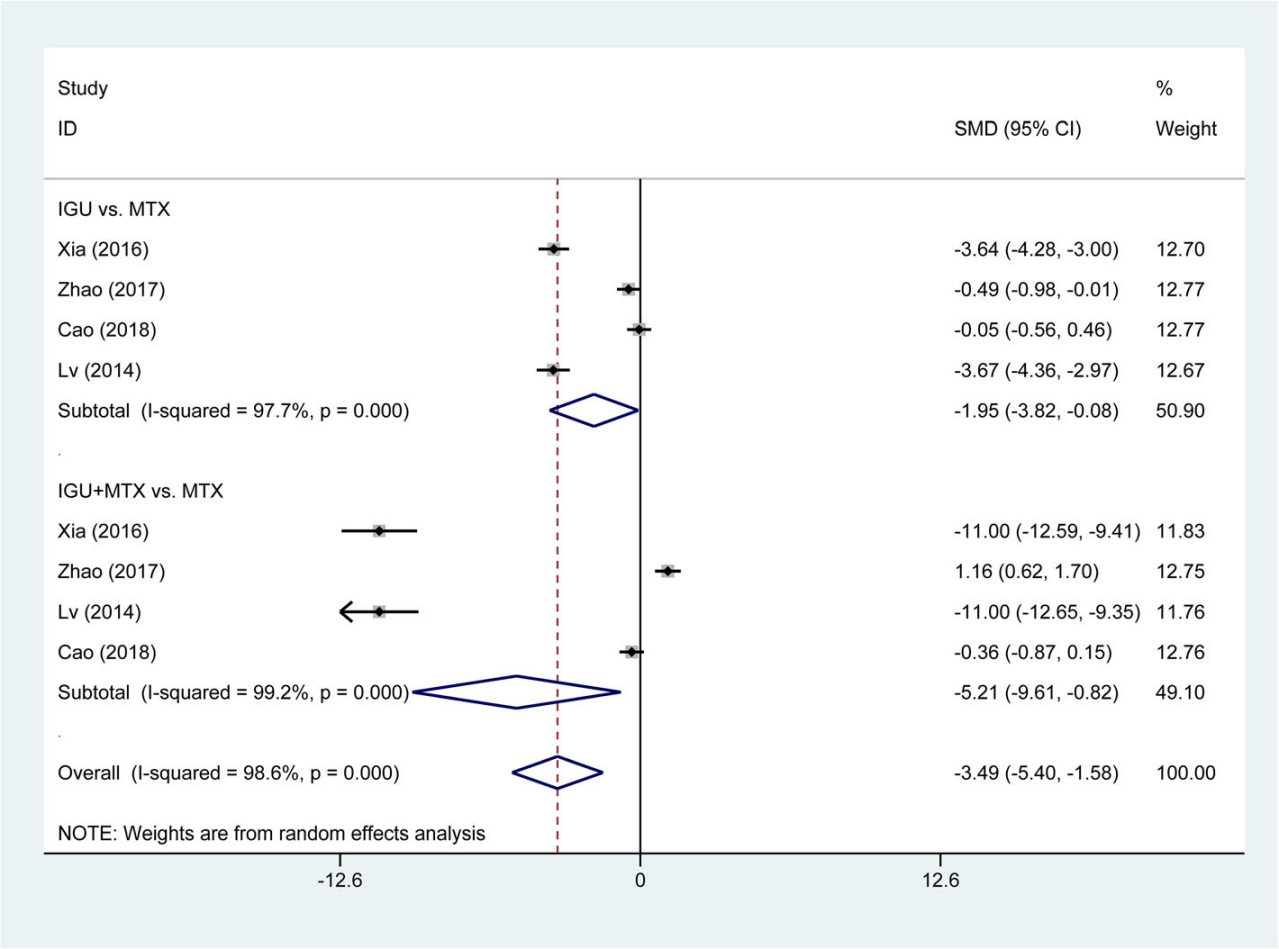
Comparison of DAS28-CRP between IGU therapy and other DMARDs monotherapy (primarily comprising MTX). IGU, iguratimod; MTX, methotrexate; DMARDs, disease-modifying antirheumatic drugs

**Fig. 5:**
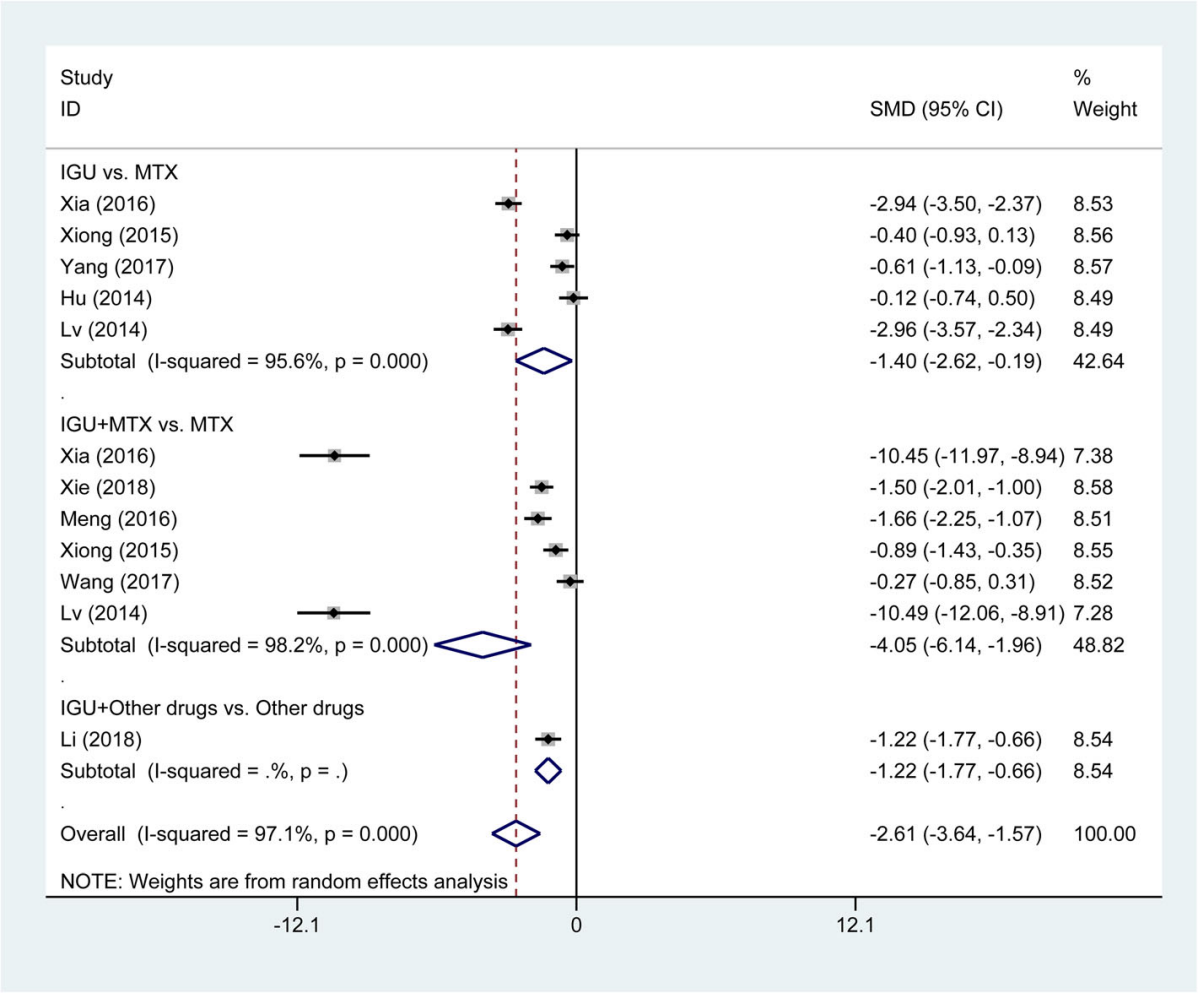
Comparison of DAS28-ESR between IGU therapy and other DMARDs monotherapy (primarily comprising MTX). IGU, iguratimod; MTX, methotrexate; DMARDs, disease-modifying antirheumatic drugs

The overall pooled analysis of ACR20 response was displayed in Fig. 3. We found that IGU therapy was associated with a significant increase of ACR20 response (OR = 1.97, 95% CI 1.29 to 3.00, *P* = 0.002), compared with the MTX monotherapy. Subgroup analysis based on different comparisons indicated that there was no statistically significant difference between IGU monotherapy and MTX monotherapy (OR = 1.19, 95% CI 0.85 to1.66, *P* = 0.322). While ACR20 response was significantly higher in patients treated with IGU plus MTX therapy compared to patients treated with MTX monotherapy (OR = 3.10, 95% CI 2.04 to 4.70, *P* < 0.001). Patients with the IGU therapy have significantly lower DAS28-CRP (SMD = −3.49, 95% CI −5.40 to −1.58, *P* < 0.001; Fig. 4) and DAS28-ESR (SMD = −2.61, 95% CI −3.64 to −1.57, *P* < 0.001; Fig. 5) than those with other DMARDs monotherapy (primarily comprising MTX). The result of subgroup analysis showed that DAS28-CRP and DAS28-ESR exhibited a marked decline in patients treated with IGU monotherapy (DAS28-CRP: SMD = −1.95, 95% CI −3.82 to −0.08, *P* = 0.041; DAS28-ESR: SMD = −1.40, 95% CI −2.62 to −0.19, *P* = 0.023); and IGU combined with MTX therapy (DAS28-CRP: SMD = −5.21, 95% CI −9.61 to −0.82, *P* = 0.020; DAS28-ESR SMD = −4.05, 95% CI −6.14 to −1.96, *P* < 0.001) compared to patients treated with MTX monotherapy (Figs. 4 and 5). For the comparison of IGU plus etanercept versus etanercept monotherapy, only 1 study reported DAS28-ESR [46]. IGU plus etanercept had lower DAS28-ESR than etanercept monotherapy (SMD = −1.22, 95% CI −1.77 to −0.66, *P* < 0.001).

**Fig. 6:**
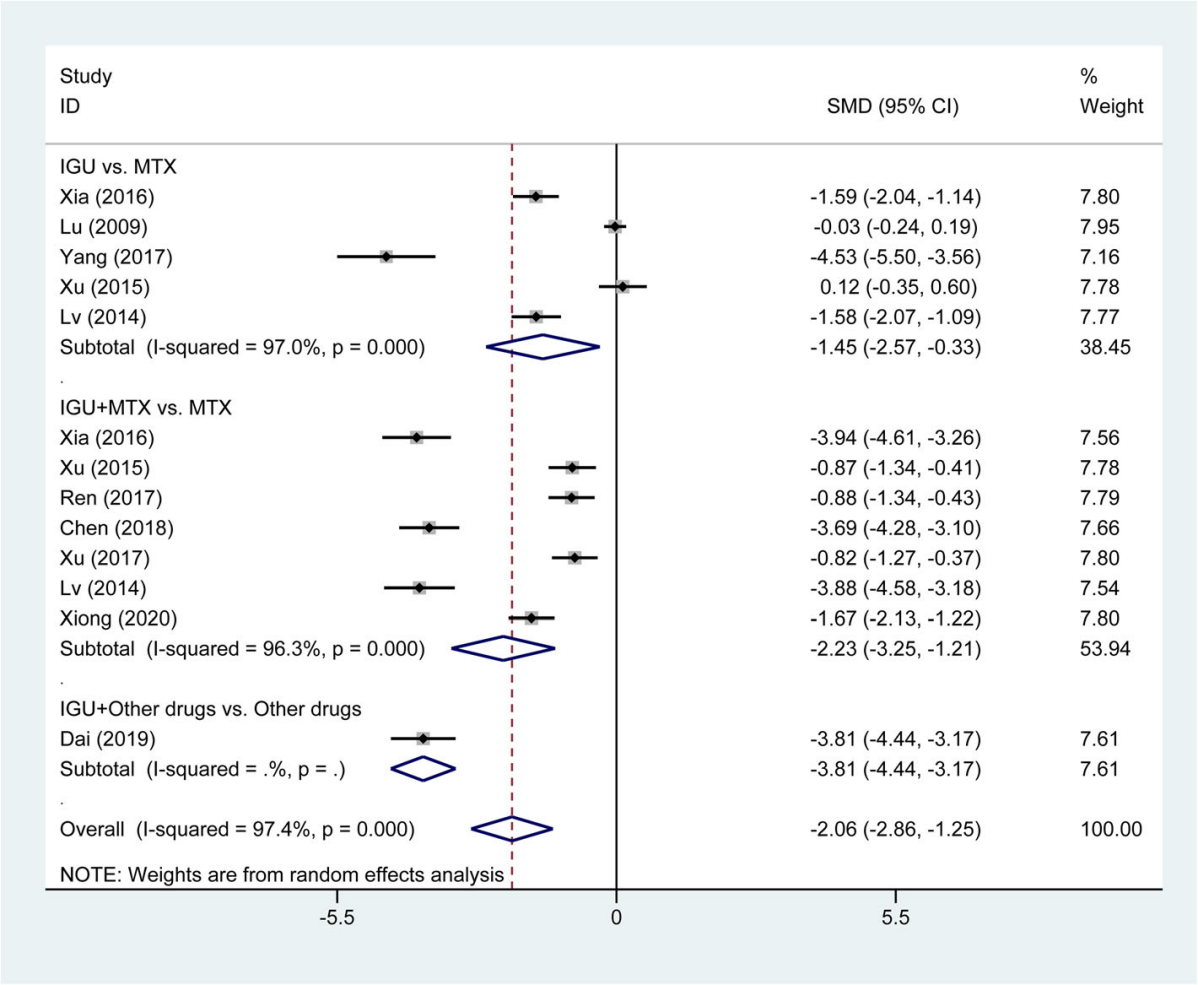
Comparison of duration of morning stiffness between IGU therapy and other DMARDs monotherapy (primarily comprising MTX). IGU, iguratimod; MTX, methotrexate; DMARDs, disease-modifying antirheumatic drugs

**Fig. 7:**
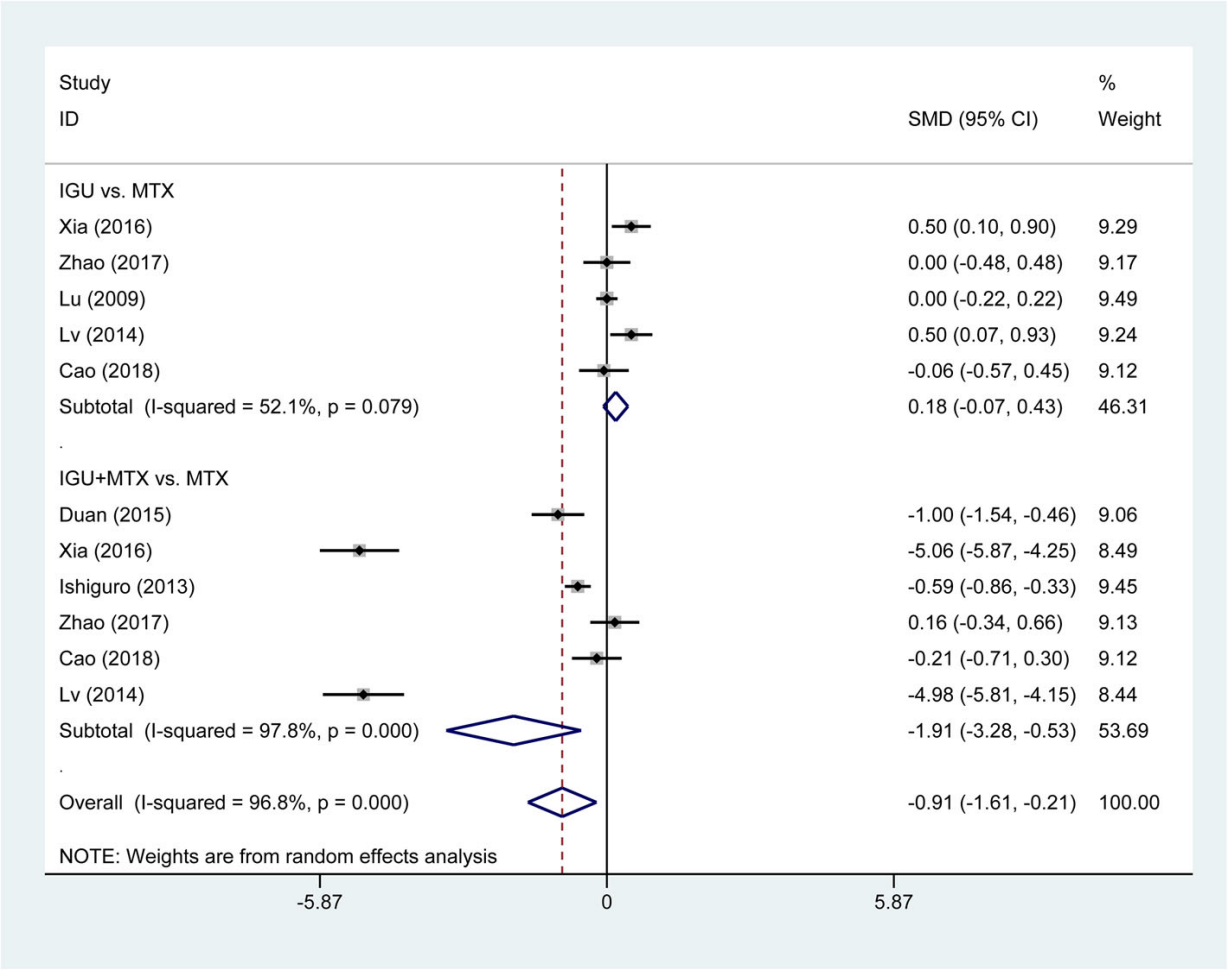
Comparison of HAQ between IGU therapy and other DMARDs monotherapy (primarily comprising MTX). IGU, iguratimod; MTX, methotrexate; DMARDs, disease-modifying antirheumatic drugs

Further analyzing other related symptoms of RA, we found that treatment of IGU therapy significantly reduced the duration of morning stiffness (SMD = −2.06, 95% CI −2.86 to −1.25, *P* < 0.001; Fig. 6) and the HAQ score (SMD = −0.91, 95% CI −1.61 to −0.21, *P* = 0.011; Fig. 7). In the subgroup analysis, both IGU monotherapy (SMD = −1.45, 95% CI −2.57 to −0.33, *P* = 0.011) and IGU combined MTX therapy (SMD = −2.23, 95% CI −3.25 to −1.21, *P* < 0.001) significantly shortened the duration of morning stiffness compared with MTX monotherapy (Fig. 6). The treatment of IGU plus leflunomide also significantly decreased the duration of morning stiffness compared with leflunomide monotherapy (SMD = −3.81, 95% CI −4.44 to −3.17, *P* < 0.001). IGU monotherapy had similar reduction in HAQ score (SMD = 0.18, 95% CI −0.07 to 0.43, *P* = 0.155), while IGU combined MTX therapy significantly decreased HAQ score compared with MTX monotherapy (SMD = −1.91, 95% CI −3.28 to −0.53, *P* = 0.007; Fig. 7).

### Adverse events

We combined data for adverse events including gastrointestinal reactions, leucopenia, increment in transaminase, increase of ALT, liver damage, and other adverse reactions. The meta-analysis of these adverse events was summarized in Table 2. Compared with MTX monotherapy, IGU monotherapy had comparable incidence of gastrointestinal reactions (OR = 0.69, 95% CI 0.40 to 1.04, *P* = 0.070), leucopenia (OR = 0.69, 95% CI 0.34 to 1.40, *P* = 0.309), increment in transaminase (OR = 2.7, 95% CI 0.38 to 19.09, *P* = 0.321), increase of ALT (OR = 0.61, 95% CI 0.38 to 1.00, *P* = 0.051), liver damage (OR = 0.13, 95% CI 0.01 to 2.61, *P* = 0.182) and fewer incidence of other adverse events (OR = 0.56, 95% CI 0.33 to 0.95, *P* = 0.032). IGU combined MTX did not increase incidence of gastrointestinal reactions (*P* = 0.921), leucopenia (*P* = 0.838), increment in transaminase (*P* = 0.193), increase of ALT (*P* = 0.985), and liver damage (*P* = 0.123), but displayed a trend of increase in other adverse reactions compared with MTX monotherapy (OR = 2.42, 95% CI 1.56 to 3.77, *P* < 0.001).

**Table 2:** The meta-analysis of the rate of adverse events of IGU monotherapy or combined therapy

Adverse reaction	Comparison	Studies, ***n***	Participants, ***n***		OR (95% CI), ***P*** value	Test of heterogeneity Cochran ***Q***, df, ***P*** value, ***I***^**2**^
	(Drug 1 vs. Drug 2)		Drug 1	Drug 2		
Gastrointestinal reactions	IGU vs. MTX	7	356	359	OR = 0.69 (0.40, 1.04), *P*= 0.070	Cochran Q = 1.46, df = 6, *P* = 0.962, *I*^2^ = 0.0%
	IGU+MTX vs. MTX	13	540	534	OR = 0.98 (0.64, 1.51), *P* = 0.921	Cochran *Q* = 5.72, df = 12, *P* = 0.929, *I*^2^ = 0.0%
	IGU+Other drugs vs. Other drugs	2	83	84	OR = 1.43 (0.28, 7.45), *P* = 0.670	Cochran *Q* = 0.36, df = 1, *P* = 0.547, *I*^2^ = 0.0%
Leucopenia	IGU vs. MTX	8	406	409	OR = 0.69 (0.34, 1.40), *P* = 0.309	Cochran *Q* = 3.39, df = 7, *P* = 0.846, *I*^2^ = 0.0%
	IGU+MTX vs. MTX	10	408	402	OR = 0.93 (0.48, 1.80), *P* = 0.838	Cochran *Q* = 2.03, df = 9, *P* = 0.991, *I*^2^ = 0.0%
Increment in transaminase	IGU vs. MTX	2	52	58	OR = 2.7 (0.38, 19.09), *P* = 0.321	Cochran *Q* = 0.01, df = 1, *P* = 0.906, *I*^2^ = 0.0%
	IGU+MTX vs. MTX	5	204	201	OR = 1.90 (0.72, 4.99), *P* = 0.193	Cochran Q = 1.12, df = 4, *P* = 0.891, *I*^2^=0.0%
	IGU+Other drugs vs. Other drugs	1	54	54	OR = 0.33 (0.01, 8.21), *P* = 0.497	Cochran *Q* = 0.0, df = 0
Increase of ALT	IGU vs. MTX	3	249	247	OR = 0.61 (0.38, 1.00), *P* = 0.051	Cochran *Q* = 1.95, df = 2, *P* = 0.376, *I*^2^ = 0.0%
	IGU+MTX vs. MTX	3	249	172	OR = 0.99 (0.50, 1.97), *P* = 0.985	Cochran *Q* = 1.08, df = 2, *P* = 0.583, *I*^2^ = 0.0%
Liver damage	IGU vs. MTX	1	30	30	OR = 0.13 (0.01, 2.61), *P* = 0.182	Cochran *Q* = 0.0, df = 0
	IGU+MTX vs. MTX	3	129	129	OR = 0.32 (0.07, 1.36), *P* = 0.123	Cochran *Q* = 0.00, df = 2, *P* = 1.000, *I*^2^ = 0.0%
Other adverse reactions	IGU vs. MTX	5	304	301	OR = 0.56 (0.33, 0.95), *P* = 0.032	Cochran Q = 1.14, df = 4, *P* = 0.888, *I*^2^ = 0.0%
	IGU+MTX vs. MTX	12	614	532	OR = 2.42 (1.56, 3.77), *P* < 0.001	Cochran *Q* = 17.74, df = 11, *P* = 0.088, *I*^2^ = 38.0%
	IGU+Other drugs vs. Other drugs	2	83	84	OR = 1.02 (0.14, 7.42), *P* = 0.986	Cochran *Q* = 0.00, df = 1, *P* = 0.986, *I*^2^ = 0.0%

*IGU* iguratimod, *MTX* methotrexate, *OR* odd ratio

### Analysis of sensitivity and publication bias

The sensitivity analysis of ACR20 showed that the removing individual studies at one time alter the overall effect slightly (upper limit of 95% CI interval lower than 3.53 in all cases, Supplementary Figure 1). The publication bias was estimated utilizing a funnel plot, which showed there was no evidence of asymmetry (Supplementary Figure 2). Furthermore, we performed an Egger test to quantify the publication bias, and the *P* value was 0.898, suggesting bias of the studies were non-existent.

## Discussion

This systematic review of 23 RCTs demonstrated that IGU, as monotherapy or combination therapy, remarkable effectiveness, and good safety in the treatment of RA. Patients receiving IGU monotherapy or combined therapy demonstrated a greater reduction in DAS28-CRP and DAS28-ESR than those with other DMARDs monotherapy (primarily comprising MTX). Regarding safety, IGU monotherapy or combined with other DMARDs therapy did not increase the risk of gastrointestinal reactions, leucopenia, increment in transaminase, increase of ALT, and liver damage compared to MTX monotherapy. Thus, taken together, these results indicated that IGU monotherapy or combined therapy may be a promising therapeutic strategy for RA.

### Efficacy

IGU has been shown to display a comparable efficacy to MTX on RA amelioration when used as monotherapy. A phase III study comparing 2 initial doses of IGU to MTX in 489 RA patients revealed that IGU 50 mg/day was equivalent to methotrexate in terms of ACR20 response (63.8% vs. 62.0%) [18]. A recently published meta-analysis of 12 trials by Sajan Shrestha [24] demonstrated that IGU monotherapy has similar ACR 20 and HAQ, and better disease state, lower CRP level and ESR compared with other DMARDs therapy, which is basically consistent with our findings, indicating that IGU may be considered a potential alternative to MTX to treat RA. In clinical practice, combination of multiple antirheumatic drugs is usually required for RA treatment, since that the combination of IGU and other DMARDs, such as MTX, etanercept, and leflunomide have synergic efficacy for RA treatment. Our results showed that IGU plus MTX was more effective than the MTX monotherapy. A study by Hara et al. implied that improvement of IGU plus MTX therapy in ACR20 response could sustain through 52 weeks, and HAQ at week 52 significantly improved compared with the values at week 24 in the patients with active RA [48]. IGU combined etanercept (a biologic agent) [46] or leflunomide (an immunosuppressive agent) [45] was demonstrated to improve functional ability, and disease status of patients with RA at 12 weeks in terms of DAS 28-ESR.

### Pharmacological mechanism

From a mechanistic view, therapeutic effects of IGU could be traced back to its anti-inflammatory, immunological action, and anabolic effects on bone metabolism. A preclinical study by Luo et al. found that the intervention of IGU plus MTX could remarkably inhibit infiltration of inflammatory cells into the synovium, and suppressed production of cytokines (IL-17, IFN-γ, IL-6, and TNF-α) and antibodies (IgG and IgG2b) in serum in the mice with collagen-induced arthritis which is widely used in preclinical studies of RA [49]. Ishiguro et al. also revealed that treatment with IGU plus MTX significantly declined the rheumatoid factor (33%, *P* < 0.001), and immunoglobulins, such as IgA, IgM, and IgG levels (*P* < 0.001) at 24 weeks in patients with RA, but these measures have no significantly change in the MTX group [20]. Dai et al. [45] found that IGU combined with leflunomide could significantly decreased serum inflammatory cytokines (TNF-α, sTREM-1), and increase the level of bone metabolic markers (25(OH)D and TPINP) in patients of RA compared with etanercept monotherapy (*P* < 0.05). Therefore, IGU may exert its clinical effect through an anti-inflammatory action, immunomodulatory action, and osteoprotective action in RA treatment.

### Adverse events

The data of post-marketing surveillance involving 2666 patients showed that long-term treatment (52-week) with IGU resulted in a tolerable safety profile in patients with RA [50]. The incidences of serious AEs, and serious adverse drug reactions were 7.35% and 4.58%, respectively. The adverse drug reactions appeared at approximately 4 weeks of treatment. Elevation of liver enzymes has been reported as a common adverse drug reaction of IGU. Treatment with IGU monotherapy (50 mg/day for 24 weeks or 25 mg/day for the first 4 weeks and 50 mg/day for the subsequent 20 weeks) result in fewer patients with increases of ALT in IGU group than in the MTX group (13.5% or 6.1% vs 23.9%, *P* < 0.05) [18]. Increases of ALT and AST were indicated to be similar at 24 weeks between combination therapy of IGU and MTX and MTX monotherapy (5.5% vs 8.0% and 9.8% vs 5.7%) [20]. This increase of liver enzymes was temporary, and patients would to recover during IGU treatment. Baseline liver dysfunction and low body weight were reported to be risk factors of liver dysfunction during IGU treatment [50]. In summary, IGU had a superior safety profile to MTX, and IGU combination did not increase incidence of AEs, which may be explained by multi-target mechanism of IGU. IGU targets upstream and downstream effectors of RA-related pathways, and did not effectively target a particular molecule. Thus, when IGU is used alone or in combination with MTX or other drugs to treat RA, its adverse events are tolerable, but patients still need to be closely monitored.

### Limitations

The limitations of this study are as follows: (1) most RCTs included do not describe the details such as allocation concealment and blind method, and there may be bias in implementation and measurement; (2) at present, the clinical data are mainly from China and Japan, and there is a lack of population from other countries; (3) the included studies reported ACR20, DAS28, etc. which may be are approximations of disease progress. Some measures which can more exactly indicate the status of disease progress need to be developed. Therefore, it is recommended that multi-center, large-scale, strictly designed, randomized, double-blind clinical studies should be performed, and the data of international clinical studies should be collected in order to better evaluate the therapeutic effect of IGU.

## Conclusion

Our analysis showed that the efficacy and safety of IGU in RA treatment was similar to that of MTX, and when combined with MTX or other DMARDs, the addition of IGU would have more benefits in terms of ACR20, DAS28-CRP, DAS28-ESR, and the duration of morning stiffness. Therefore, IGU has good efficacy and tolerance, and is a promising drug in clinical practice. IGU may be regarded as a potential alternative to MTX, and also a preferable choice when combined with other DMARDs for the treatment of RA. Large-scale and high-quality RCTs are necessary to further confirm our findings.

## Supplementary Information

**Additional file 1: Supplementary figure 1.** Meta-analysis random-effects estimates (linear form)

**Additional file 2: Supplementary figure 2.** Funnel plot with pseudo 95% confidence limits

## Data Availability

Not applicable.
