# Supplementary figures and images for: Effectiveness of iguratimod as monotherapy or combined therapy in patients with rheumatoid arthritis: a systematic review and meta-analysis of RCTs
Source: J Orthop Surg Res. 2021 Jul 16;16:457. doi: 10.1186/s13018-021-02603-2 (PMC8283838; doi:10.1186/s13018-021-02603-2)

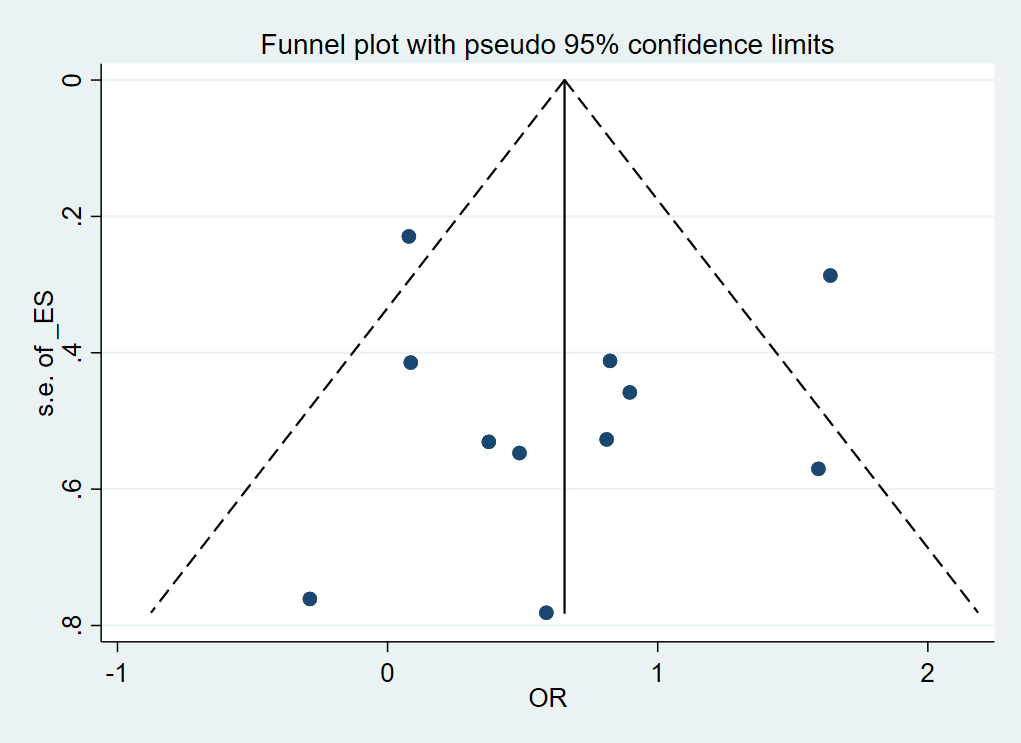

Supplement: Supplementary file 2 — Additional file 2: Supplementary figure 2. Funnel plot with pseudo 95% confidence limits [file 13018_2021_2603_MOESM2_ESM.tif]
